# A Case Series Analysis of Dental Extractions’ Outcome in Cats with Chronic Gingivostomatitis Carrying Retroviral Disease

**DOI:** 10.3390/ani11113306

**Published:** 2021-11-19

**Authors:** Marta Silva, Marta Fernandes, Mónica Fialho, Lisa Mestrinho

**Affiliations:** 1 CIISA, Centro de Investigaҫão Interdisciplinar em Sanidade Animal, Faculdade de Medicina Veterinária, Universidade de Lisboa, Av. da Universidade Técnica, 1300-477 Lisbon, Portugal; lisamestrinho@gmail.com; 2 Hospital VetCentral, Rua António de Andrade, Lote 1141, 2820-287 Almada, Portugal; martafreixof@gmail.com; 3 Instituto de Saúde Ambiental (ISAMB), Faculdade de Medicina, Universidade de Lisboa, Av. Prof. Egas Moniz, 1649-028 Lisbon, Portugal; monicafialho15@gmail.com

**Keywords:** feline chronic gingivostomatitis, feline immunodeficiency virus, feline leukaemia virus, dental extractions, postsurgical outcome

## Abstract

**Simple Summary:**

Feline chronic gingivostomatitis (FCGS) is a chronic, painful, oral inflammatory disease, which can be associated with retroviral disease comorbidity’s, namely feline immunodeficiency virus (FIV) and feline leukaemia virus (FeLV). A total 111 case series of cats affected by this oral disease, treated with dental extractions, were analyzed retrospectively, considering if they carried or not one of these retroviral diseases. Cats with lingual ulcers, independently from their retroviral status, were 2.7 times more prone to have a worse response to dental extractions than cats without lingual ulcers. When compared with cats without retroviral disease, FeLV-positive cats presented less proliferative lesions and tended to have more lingual ulcers. Furthermore, these cats had a significantly worse outcome, with 7.5 times more chances of having no improvement.

**Abstract:**

This study aims to evaluate and compare the clinical outcome after dental extractions of cats with FCGS infected with feline immunodeficiency virus (FIV) and feline leukaemia virus (FeLV). A retrospective case series included cats with diagnosis of FCGS, availability of detailed clinical records, full-mouth dental radiographs, and retroviral disease test results. Effectiveness of surgical treatment (EOT) was registered. Three groups were defined: control, FIV and FeLV. In this study, 111 cats were included: 60 controls, 29 FIV- and 22 FeLV-positive cats. When compared with control cases, FeLV-positive cats had significantly less proliferative stomatitis lesions, and they tended to have more lingual ulcers. Concurrently, FeLV-positive cats had significantly less tooth resorptive lesions. No other significant differences in FCGS clinical signs were found between groups. FeLV-positive cats had a significantly worse outcome after dental extractions compared to the other groups. In fact, FeLV-positive cats had 7.5 times more chances of having no improvement after dental extractions. This study concludes that the response to dental extractions in FeLV-positive cats is significantly worse, when comparing to cats that do not carry retroviral disease. Therefore, it is important to acknowledge the effect of FeLV status on the prognosis of these cats.

## 1. Introduction

Feline chronic gingivostomatitis (FCGS) is a painful oral inflammatory disease, which can lead to severe malnutrition and dehydration in critical cases [1,2,3,4,5]. Reported prevalence in 4858 cats, during a 12-week period, was 0.7%, in North West England first opinion practices [5]. FCGS lesions present typically as ulcerative and/or proliferative lesions characterized by a symmetrical and bilateral pattern, of friable consistency, bleeding easily when manipulated [2,6,7,8]. Histopathological results show a lymphoplasmacytic infiltrate, indicative of the chronic inflammatory process. Polyclonal hypergammaglobulinemia is also a consistent finding [1,6,7,9,10,11,12].

FCGS is considered multifactorial, although recent evidence suggests a T-cell disfunction [12], related to infectious and non-infectious causes, such as viruses, dental plaque, hypersensitivity reactions, environment conditions and stress in multi-cat households [6,7,8,13,14,15,16,17,18,19]. With regards to viral agents, feline calicivirus (FCV) appears to carry out a relevant role in FCGS, three times more prevalent in these animals when compared to the general cat population [2,15]. However, studies have not been able to consistently prove that chronic infection by FCV is directly implicated in the pathogenesis of FCGS [2,8,13,14].

Treatment response and long-term effectiveness in all FCGS cases is still inconsistent [2,9]. Its main goal is to reduce oral inflammation and antigen load, to re-establish the oral balance and to reduce pain [2]. The main treatment strategies are surgical and medical, often combined. The first one has demonstrated the best long-term outcome, with full-mouth (FME) or partial-mouth extractions (PME) when including premolar and molar teeth only [2,20]. Approximately 80% of the cats submitted to dental extractions, FME or PME, obtained significant improvement, with some achieving complete remission of the clinical signs, with or without the need for combined medical treatment [21,22,23].

Considering FCGS’s strong immune-mediated basis, medical approaches have mainly consisted of drugs with immunosuppressive and/or immunomodulatory properties, namely glucocorticoids [2,24,25,26,27], cyclosporine [26,27,28,29,30] and feline recombinant interferon omega [31,32,33,34,35,36,37,38,39]. More recently, mesenchymal stem cells were reported as a new therapeutic approach [11,40,41].

Feline immunodeficiency virus (FIV) and feline leukaemia virus (FeLV) are two important infectious agents in cats and are known to cause immune and inflammatory disfunction and/or immunosuppression with increased risk of opportunistic infections [35,42,43,44]. Their association with FCGS is still not completely elucidated, but both viruses may act as aggravating factors [1,2,14,42,45].

It is not known whether cats carrying retroviral disease and FCGS would respond differently to surgical treatment. Therefore, this study aims to evaluate and compare the postsurgical outcome of FCGS patients with and without retroviral disease.

## 2. Materials and Methods

### 2.1. Study Design and Variables

This study was retrospectively designed, which included cats with diagnosis of FCGS from 4 veterinary referring practices, followed for a minimum of 3 months, between 1 January 2010 and 30 April 2021. All cases were diagnosed and managed by one of the authors [blinded for review], in collaboration with the referral practitioners. Selection criteria included a complete clinical history, pre- and postoperative full-mouth dental radiographs, and retroviral disease test results. All cases were managed under the highest standard of good care, which included the treatment with FME or PME and adjuvant medical support (when needed) to achieve remission of clinical signs. Informed consent was obtained from all cat owners.

Three groups were defined: control (all cats FIV and FeLV-negative), FIV (all cats FIV-positive but FeLV-negative) and FeLV (all cats FIV-negative but FeLV-positive). Criteria used to assign cats into the groups was solely retroviral status. Only cats with conclusive results (in-house and laboratory immunoassay) were included in each group. All cats were tested or retested at the same laboratory using the same laboratory technique, to confirm their FIV/FeLV status by Enzyme Linked Immunoabsorbent Assay (ELISA). For FIV: ViraCHECK/FIV, Synbiotics—sensitivity ≥92.6% (95% CI 82.4–97.1) and specificity ≥99.8 (95% CI 98.8–100). For FeLV: ViraCHECK/FeLV, Synbiotics—sensitivity ≥94.9% (95% CI 83.1–98.6) and specificity ≥98.4% (95% CI 96.8–99.2). Animals that did not comply with inclusion criteria were excluded from the study. When needed, objective information regarding time of FCGS diagnosis and the MTBS (medical treatment before surgery) performed by each animal was completed, by e-mail or phone questionnaire with the assistant clinician.

Information from each cat included: breed; sex; reproductive status; age group at the time of dental extractions—juvenile (<3 years), adult (≥3 to <7 years), senior (≥7 to <10 years) and geriatric (≥10 years); date of the first dental surgery; time between FCGS diagnosis and dental extractions: “<1 year” and “≥1 year”; outdoor access; adoption from a cattery establishment; FIV and FeLV status; FCV status (performed using RT-PCR); oral biopsy; lesion pattern—buccal/alveolar stomatitis, lingual ulcers, caudal stomatitis, proliferative stomatitis, ulcerative stomatitis; and radiographic findings—tooth resorption (TR), periodontal disease and retained roots; number of lesions “≤4” or “≥5” (buccal stomatitis, alveolar stomatitis, lingual ulcers, caudal stomatitis, periodontal disease, retained roots and/or TR); medical treatment prescribed before surgery (MTBS)—analgesic opioids, non-steroidal anti-inflammatory drugs (NSAIDs), antiseptic oral gel, corticosteroids, human recombinant interferon alfa (rHuIFN-α) and feline recombinant interferon omega (rFeIFN-ω); when applicable, the duration and dosage of corticosteroid therapy: “<1 month”, “between 1 month and 3 months” e “>3 months”; type of dental surgery—FME or PME, and if performed in one phase or two phases; need for medical treatment after surgery (MTAS); occurrence of relapse of clinical signs or, for the contrary, the occurrence of clinical cure; effectiveness of treatment (EOT) final score (at least 3 months after the performance of dental extractions); if applicable, the date and cause of death.

Oral biopsies, when performed, at least three pieces of tissue were collected in the most representative locations of FCGS: gingiva, caudal and vestibular mucosa. Due to increased post-biopsy morbidity, the tongue was not biopsied.

MTBS was registered if medication was being given at least one week before surgery. MTAS was registered if medication was given 15 days after normal postsurgical medical treatment. It was only collected information about the need for MTAS, it was not specified which drugs were used. Relapse cases were also registered. Relapse was considered when clinical signs returned 2 months or more after surgery.

EOT final score (Table 1) was defined as previously described [23], determined at least after 3 months after dental extractions. Finally, EOT score was grouped into two categories: “no significant improvement” vs. “significant improvement or cure”, corresponding to the grouping of EOT 0 and 1 vs. EOT 2 (a and b) and 3 (a and b).

### 2.2. Statistical Analysis

Data registration, exploratory and descriptive analysis were performed using a commercial software (IBM SPSS Statistics for Windows (2019). IBM Corp. Released 2019. Version 26.0. Armonk, NY: IBM Corp). For inferential statistics analysis, R Software version 3.6.3 (R Core Team (2020). R: A language and environment for statistical computing. R Foundation for Statistical Computing, Vienna, Austria) was used. Pairwise comparisons of groups of animals (control, FIV and FELV) were performed using Fisher’s Exact Test. After Bonferroni correction, *p* ≤ 0.017 was considered statistically significant. To explore the effects of FCGS clinical presentation (lingual ulcers, proliferative stomatitis, TR and periodontal disease) and treatment variables (use of corticosteroids, surgery and number of phases) in clinical response, simple and multiple binary logistic regression models were used, considering “no significant improvement” as the event of interest. Model 1 presents crude Odds Ratio (OR) and respective 95% Confidence Interval (IC) as a measure of association between each independent variable and the outcome variable. In the Model 2, the effect of the group was controlled for clinical relevant variables, whereas Model 3 was adjusted for group and treatment variables. Nagelkerke pseudo R^2^ is given as indicator of model fit. A significance level of 5% was assumed for each parameter. Finally, it was performed a survival analysis of the three groups, by the performance of Kaplan-Meier curves and log-rank analysis.

## 3. Results

### 3.1. Sample Characterization, Clinical and Treatment Variables

One-hundred and eleven cats were included in this study, 60 in control group, 29 in group FIV and 22 in group FeLV. Ninety-six percent (107/111) of cats were Domestic Shorthair. The average of postoperative follow-up for control, FIV and FeLV groups was 1206 ± 890, 971 ± 572 and 866 ± 745 days, respectively. Mean age at time of tooth extraction of control, FIV and FeLV groups was 7 ± 4, 7 ± 3 and 6 ± 3 years, respectively. Sex, age group, outdoor access, cattery origin, neutered status, FCV results and time between diagnosis and surgery for all groups are shown in Table 2. No differences were found between groups for the variables mentioned. In FeLV, FIV and control groups, 9%, 10% and 2% were not neutered cats, with no significant differences found between groups.

Histopathological evaluation was performed in 40% (24/60) of cats of group control, 21% (6/29) of group FIV and in 5% (1/22) of group FeLV, all confirmed an inflammatory lympho-plasmocytic infiltrate, typical of the chronic process.

FIV-positive cats had significantly less caudal stomatitis than cats of control group (Table 3). Cats of group FeLV had significantly less proliferative stomatitis and less TR than control cases. Of note, it was possible to observe that cats FeLV-positive presented more lingual ulcers than cats of group control, although not significantly (Table 3).

Medical approach prior to surgery was significantly less prescribed in cats from group FIV than control cases (Table 4). Also, cats FeLV-positive had significantly less oral gel as MTBS, compared to control and FIV groups (Table 4). In total, the most prescribed treatment was NSAID. Corticosteroids given included methylprednisolone and prednisolone—prescribed orally at a dosage of 1 to 2 mg/kg once a day. Methylprednisolone acetate was also administered subcutaneously at the same dose, every month. Time of corticosteroid therapy was not significantly different between groups (results not shown). More than half of the cats in each group had received more than 3 months of corticoid therapy before surgery—54% (15/28) in group control, 62% (8/13) in group FIV and 64% (7/11) in group FeLV.

### 3.2. Response to Treatment

Time between diagnosis of FCGS and surgery was more than 1 year in 53% (32/60), 66% (19/29) and 77% (17/22) of cats of group control, FIV and FeLV, respectively. This time interval was not significantly associated with the response to surgical treatment, for each of the three groups (results not shown). Since the dosage was similar in all cats treated with corticosteroids, the dose variable was not analyzed. Due to the reduced number of cats treated with methylprednisolone acetate, analysis was not performed either. Table 5 summarizes the treatment response variables registered in this case series. MTAS and disease relapse were not significantly different between groups.

Cats from FeLV group had significantly worst response to dental extractions when compared to control cases. FeLV-positive cats had 7.5 times more chances of having no improvement with dental extractions when compared to control group (Table 6). Cats with lingual ulcers, independently of which group they were in, were 2.7 times more prone to have a worse response to surgery than cats without lingual ulcers (Table 6).

### 3.3. Postsurgical Survival Analysis

**Figure 1:**
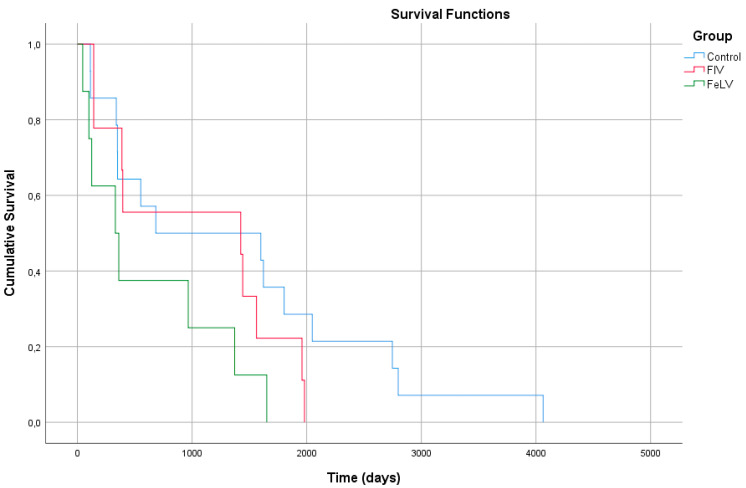
Kaplan-Meier curves from 16 cats of group control, 9 cats from group FIV and 8 cats from group FeLV.

Until the endpoint of data research, the percentage of deaths registered were: 27% (16/60) at control group, 31% (9/29) at FIV group and 36% (8/22) at FeLV group. Mean age at time of death as well as mean and median postoperative survival time are presented in Table 7. No significant differences were found between FIV, FeLV and control Kaplan-Meier curves (Figure 1), by log-rank analysis (*p* = 0.085). Excluding unknown causes of death, main cause of death in control cases was chronic renal disease (CRD) and secondly lymphoma. In FIV group main cause of death was also CRD and in FeLV group most cats died with FeLV-related diseases, such as anaemia and lymphoma.

## 4. Discussion

The main goal of this study was to evaluate and compare the postsurgical outcome of cats with FCGS, carrying or not retroviral disease. It was possible to identify some risk factors associated with a worse response to dental surgery and to observe some clinical differences, especially in FeLV-positive cats.

There was no significant difference in sex and in reproductive status distribution between the study groups. However, according to prior studies, FIV and FeLV-positive cats were mainly sexually intact, especially male cats, since they were more prone to carry FIV antibodies and FeLV antigens [9,46]. These observations could be the result of an overrepresentation of intact cats, possibly cats with outdoor access, and/or cats from a cattery. Age average at dental extractions was similar between the three groups and to what has been reported [23]. 

FCV positive status was present in more than two-thirds of the FCV-tested cases, being highly prevalent and similar between groups. Although concurrent infection with FCV, FeLV, and FIV has been associated with more severe oral lesions [13], co-infection with FCV and retroviral disease was not related to an increased susceptibility to FCV infection, since all cats had similar exposure (outdoor access and cattery origin).

It has been suggested that the sooner dental extractions are performed, the better the postsurgical outcome for cats with FCGS [23]. However, in this study, a longer time between diagnosis and surgical treatment was not significantly associated with a worse outcome, with most cats being submitted to dental extractions more than one year after the diagnosis of FCGS. This suggests some resistance from cat owners or perhaps the referring veterinarian to accept or recommend dental extractions. Even though outdoor access and catteries allow close contact with potentially infected cats [2,43], there were no significant differences between cats with and without retroviral disease, regarding these variables. 

With regards to clinical presentation, FIV-positive cats had significantly less caudal stomatitis than cats without retroviral disease. Caudal stomatitis is considered a hallmark in FCGS, but the diagnosis is based on the bilateral distribution of stomatitis lesions extending from the alveolar to the buccal, lingual, caudal, and/or palatal mucosa, not necessarily including all locations. The lack of caudal stomatitis lesions probably resulted from concurrent medical treatment at time of diagnosis, especially with corticosteroids. This fact explains the one control case and some cases with retroviral disease. In the other cases with retroviral infection, the observation of buccal/alveolar stomatitis can represent one of the first signs of systemic immunodeficiency, since first lesions can be most directly related to oral plaque. However, retroviral disease can change the evolution, distribution, and pattern of oral lesions since the CD8^+^/CD4^+^ ratio of T cells is disturbed, especially in FIV-infected cats [47]. Therefore, even in the absence of extension of inflammation to the caudal mucosa, the presence of bilateral and persistent inflammatory lesions extending from the alveolar to the buccal mucosa, associated with other chronic persistent inflammatory lesions to the tongue and/or palatal mucosa, should also be considered FCGS.

FeLV-positive cats had significantly less proliferative stomatitis and less TR than control cases. Considering that wound healing is impaired in immunosuppressed cats [48,49], and FeLV can infect fibroblast precursor cells and interfere with fibroblast function [50], this could explain why oral lesions in this group are predominantly non-proliferative. The occurrence of TR was not positively associated with cats carrying FeLV infection, which meets the observations previously reported by other authors [51,52], where age is pointed as the main risk factor to the development of TR in cats. 

Additionally, there was a tendency for FeLV-positive cats to present more lingual ulcers than cats without retroviral disease. Lingual inflammatory lesions are frequently associated with FCV infection [20,23,53] but, interestingly, in this study, only 32% (10/31) of the FCV-positive cats presented with lingual ulcers. However, due to the low number of FCV-positive cases in the FeLV group, it was not possible to support or refute the hypothesis that FeLV cats co-infected with FCV might have a worse clinical presentation (with lingual ulcers) and outcome. Other authors have suggested that immunosuppression by FeLV can lead to a predisposition and manifestation of secondary infections (by FCV, for example) [13,42,43,54], and consequently present more frequently with lingual ulcers. 

With regards to treatment variables, it was possible to notice that MTBS was significantly less prescribed to FIV-positive cats than to cats from the control group. The same tendency was observed for FeLV-positive cats, although not significantly. This is likely due to a reluctance of using immunosuppressive drugs (e.g., corticosteroids or ciclosporin) in retroviral infected cats, since these might aggravate the underlying disease. Additionally, FeLV-positive cats had significantly less oral gel prescribed as MTBS. This may be explained by the low number of cases in the FeLV group or due to some non-identified selection bias from the clinician or owner, which might have limited the use of such approach. These differences could be a result of chance, due to the limited number of cases, or could reflect a primary difficulty from owners to topically treat these cats with the oral gel.

The variables used on the simple and multiple binary logistic regression models were chosen, having in consideration the statistical differences between groups and their potential role in the postsurgical outcome. Cats with lingual ulcers, independently from their retroviral status, were 2.7 times more prone to have a worse response following dental extractions than cats without lingual ulcers. The main goal of the surgical procedure is to reduce the antigenic oral stimulation, by eliminating dental plaque [17,55]. It is possible that cats with lingual ulcers remain painful after surgery and might need additional medical treatment to reduce oral inflammation and improve tissue healing. 

Given the metabolic and immunosuppressive effects of corticosteroids [24,27,56], it should be expected that cats undergoing corticoid therapy prior to surgery, especially those bearing retroviral disease, would have a worse postsurgical outcome because of delayed tissue healing and increased predisposition to opportunistic infections, caused by a potentially compromised immune response. However, no significant associations were found between corticosteroids usage and the postoperative outcome. The interpretation of these results should be done carefully. In all cases treated with corticosteroids, the dose used was within the anti-inflammatory range and therefore seems to be relatively safe in cats, independently of their retroviral status. On the other hand, because there were no cases treated with immunosuppressive dosage of corticosteroids it was not possible to evaluate a likely unfavorable outcome in cats with retroviral disease. Regardless, an immunosuppressive dosage should not be advised in cats carrying retroviral disease and its long-term usage should be avoided due to the risk of iatrogenic complications.

In agreement with prior results [23], there was no association between FME vs. PME or the number of phases of FME and the outcome of post-dental extractions. To the author’s knowledge, the effect of the number of phases for FME was never evaluated. It is advocated that FME should be done at first attempt to achieve the best chance of cure [57]. According to the results obtained in this study, neither the number of teeth, nor the need to perform FME at first attempt, significantly changed the outcome. This suggests that the true role of dental plaque on FCGS aetiology is questionable, as was already proposed by other studies [23,30].

Dental extractions resulted in significant improvement in 90% of cats without retroviral disease, 79.3% of FIV-positive cats and 54.5% of FeLV-positive cats. The remission of FCGS observed for control cases was superior to previously published results [20,22,23]. This could be the result of a long-term follow-up, since most control cases were followed for more than 2 years. Perhaps remission can be obtained in the long-term and not just due to dental extractions alone. More studies involving the long-term follow-up of FCGS cases are needed to understand if there are other events that might contribute to a favorable outcome. 

The overall response to treatment in FCGS cats carrying retroviral disease has never been evaluated to the author’s knowledge. FIV-positive cats seem to have no significant difference in overall response compared to control cases. On the other hand, the response to FCGS treatments in FeLV-positive cats was significantly worse, with 7.5 times more chances of having no significant improvement when compared to those without retroviral disease. Retroviral positivity does not necessarily translate into disease, especially in FIV-positive cats, where the disease can remain latent for years without evolving into immunodeficiency. However, dental disease and stomatitis are considered manifestations of immunosuppression in the affected cats [47]. Therefore, we can consider that FIV and FeLV-positive cats can potentially be affected by retroviral disease and develop FCGS. If FCGS is just a result of immunosuppression it could be argued that FeLV-positive cats would be at a higher risk of developing FCGS, when compared to FIV-positive cats. This observation is also supported by the survival analysis performed in this study, since FeLV-infected cats tended to have a shorter survival time in comparison to FIV-positive and control cases. FeLV infection can have a relevant impact on the longevity and wellbeing of the affected cats since it can lead to immunosuppression [47], and consequently potentiate the occurrence of oral inflammatory lesions previously initiated by local processes [1,43,45].

Interestingly, 30% of cats in this study sample died with CRD. FCGS, similarly to other oral chronic inflammatory diseases, can be associated with systemic manifestations, as a consequence of the increased inflammatory burden [58], increasing the risk for CRD secondary to immune complex glomerulonephritis [59,60,61]. However, because CRD is not necessarily a consequence of oral disease and is a common cause of death in cats [62], we cannot directly correlate, based on our results, the deaths caused by CRD with FCGS. It would be important to perform a large cohort study of cats with FCGS, in order to evaluate its systemic impact in the long-term, comparing to cats without oral disease.

Limitations of this study were mostly related to its retrospective nature, a long timeframe of 10 years and a low number of cases. Additional information related to MTBS and time to surgery was collected by veterinary inquiry. MTBS was based on the interpretation of clinical records given by the referral clinician and therefore interpretation bias could be a concern. Also, the time between diagnosis and surgery was a crude categorization, which could be improved in order to obtain more accurate results. False positive or false negative results in the retroviral and FCV tests performed could also cause bias, although the inclusion criteria tried to include only conclusive results. Some treatments, namely ciclosporin, interferon or even oral gel, were not extensively used in the studied population. Therefore, their impact was not fully appreciated.

## 5. Conclusions

Retroviral disease status for FeLV has a significant effect on the outcome of treatment of FCGS. Therefore, it is important to acknowledge its influence in FCGS severity and prognosis.

## Figures and Tables

**Table 1:** EOT score for FCGS surgical treatment.

EOT Score for FCGS Treatment		
0		no improvement or worsening of clinical signs following FME or PME with continuing MTAS at final recheck examination
1		little improvement with ongoing clinical signs after FME or PME and continuing MTAS at final recheck examination
2		substantial improvement with ongoing but improved clinical signs
	a	substantial improvement after dental surgery without the need for MTAS until final recheck examination
	b	substantial improvement after dental surgery, although MTAS was necessary for some finite period of time until final recheck examination
3		complete resolution of clinical signs after FME or PME: clinical cure
	a	complete resolution following FME or PME without the need for MTAS until final recheck examination
	b	complete resolution following FME or PME, although MTAS was necessary for some finite period of time until final recheck examination

**Table 2:** General characterization of the retrospective series.

	Total	Control	FIV	FeLV	
	*n* (%)	*n* (%)	*n* (%)	*n* (%)	*p* Value
**Total Sample**	111 (100%)	60 (54%)	29 (26%)	22 (20%)	
**Sex**					0.456
Female	51 (46%)	31 (52%)	11 (38%)	9 (41%)	
Male	60 (54%)	29 (48%)	18 (62.1%)	13 (59%)	
**Age Group**					0.593
Junior	12 (11%)	9 (15%)	1 (3%)	2 (9%)	
Adult	51 (46%)	25 (42%)	14 (48%)	12 (55%)	
Senior	24 (22%)	11 (18%)	8 (28%)	5 (23%)	
Geriatric	24 (22%)	15 (25%)	6 (21%)	3 (14%)	
**Outdoor Access**					0.295
No	17 (15%)	12 (20%)	2 (7%)	3 (14%)	
Yes	94 (85%)	48 (80%)	27 (93%)	19 (86%)	
**Cattery (Origin)**					0.592
No	78 (70%)	42 (70%)	22 (76%)	14 (64%)	
Yes	33 (30%)	18 (30%)	7 (24%)	8 (36%)	
**Neutered**					0.117
No	6 (5%)	1 (2%)	3 (10%)	2 (9%)	
Yes	105 (95%)	59 (98%)	26 (90%)	20 (91%)	
**FCV**					0.426
Not tested	73 (66%)	35 (58%)	20 (69%)	18 (82%)	
Negative result	7 (6%)	4 (7%)	3 (10%)	0 (0%)	
Positive result	31 (28%)	21 (35%)	6 (21%)	4 (100%)	
**Time between Diagnosis and Surgery**					0.134
<1 year	43 (39%)	28 (47%)	10 (34%)	5 (23%)	

≥1 year	68 (61%)	32 (53%)	19 (66%)	17 (77%)	

**Table 3:** Clinical presentation and radiographic findings variables.

	Total	Control	FIV	FeLV			*p* Value	
	*n* (%)	*n* (%)	*n* (%)	*n* (%)	*p* Value	FIV vs. Control	FELV vs. Control	FIV vs. FELV
**No. of Lesions**					0.147			
≤4	53 (48%)	24 (40%)	15 (52%)	14 (64%)		0.364	0.080	0.569
≥5	58 (52%)	36 (60%)	14 (48%)	8 (36%)				
**Buccal/Alveolarstomatitis**					0.113			
No	5 (5%)	1 (2%)	3 (10%)	1 (5%)		0.100	0.467	0.625
Yes	106 (95%)	59 (98%)	26 (90%)	21 (95%)				
**Lingual Ulcers**					0.143			
No	80 (72%)	46 (77%)	22 (76%)	12 (55%)		1.000	0.061	0.140
Yes	31 (28%)	14 (23%)	7 (24%)	10 (45%)				
**Caudal Stomatitis**					0.020 ^#^			
No	8 (7%)	1 (2%)	5 (17%)	2 (9%)		0.013 *	0.174	0.684
Yes	103 (93%)	59 (98%)	24 (83%)	20 (91%)				
**Proliferative Stomatitis**					0.013 ^#^			
No	59 (53%)	25 (42%)	17 (59%)	17 (77%)		0.175	0.006 *	0.233
Yes	52 (47%)	35 (58%)	12 (41%)	5 (23%)				
**Tooth Resorption**					0.022 ^#^			
No	30 (27%)	11 (18%)	8 (28%)	11 (50%)		0.408	0.010 *	0.145
Yes	81 (73%)	49 (82%)	21 (72%)	11 (50%)				
**Periodontal Disease**					0.271			
No	28 (25%)	19 (32%)	5 (17%)	4 (18%)		0.205	0.278	1.000
Yes	83 (75%)	41 (68%)	24 (83%)	18 (82%)				
**Retained Roots**					0.873			
No	59 (53%)	31 (52%)	15 (52%)	13 (59%)		1.000	0.622	0.777
Yes	52 (47%)	29 (48%)	14 (48%)	9 (41%)				

* p ≤ 0.017 was considered significant after Bonferroni correction for multiple comparisons. ^#^ *p* ≤ 0.05.

**Table 4:** Treatment variables.

	Total	Control	FIV	FeLV			*p* Value	
	*n* (%)	*n* (%)	*n* (%)	*n* (%)	*p* Value	FIV vs. Control	FELV vs. Control	FIV vs. FELV
**MTBS**					0.006 ^#^			
No	6 (5%)	0 (0%)	4 (14%)	2 (9%)		0.010 *	0.070	0.688
Yes	105 (95%)	60 (100%)	25 (86%)	20 (91%)				
**Analgesic Opioids**					0.370			
No	43 (39%)	20 (33%)	12 (41%)	11 (50%)		0.487	0.203	0.581
Yes	68 (61%)	40 (67%)	17 (59%)	11 (50%)				
**NSAIDs**					0.407			
No	35 (32%)	16 (27%)	10 (34%)	9 (41%)		0.465	0.280	0.772
Yes	76 (68%)	44 (73%)	19 (66%)	13 (59%)				
**Oral Gel**					0.007 ^#^			
No	53 (48%)	23 (38%)	13 (45%)	17 (77%)		0.647	0.002 *	0.025 ^#^
Yes	58 (52%)	37 (62%)	16 (55%)	5 (23%)				
**Corticosteroids**					0.905			
No	57 (51%)	32 (53%)	14 (48%)	11 (50%)		0.821	0.808	1.000
Yes	54 (49%)	28 (47%)	15 (52%)	11 (50%)				
**rHuIFN-α**					0.146			
No	104 (94%)	58 (97%)	25 (86%)	21 (95%)		0.085	1.000	0.375
Yes	7 (6%)	2 (3%)	4 (14%)	1 (5%)				
**rFeIFN-ω**					0.403			
No	97 (87%)	52 (87%)	24 (83%)	21 (95%)		0.750	0.433	0.218
Yes	14 (13%)	8 (13%)	5 (17%)	1 (5%)				
**Surgery**					0.763			
PME	53 (48%)	30 (51%)	14 (48%)	9 (41%)		1.000	0.463	0.777
FME	57 (52%)	29 (49%)	15 (52%)	13 (59%)				
**No. of Phases**					0.437			
1	97 (87%)	50 (83%)	27 (93%)	20 (91%)		0.323	0.499	1.000
2	14 (13%)	10 (17%)	2 (7%)	2 (9%)				

* p ≤ 0.017 was considered significant after Bonferroni correction for multiple comparisons. ^#^ *p* ≤ 0.05.

**Table 5:** Response to treatment.

	Total	Control	FIV	FeLV			*p* Value	
	*n* (%)	*n* (%)	*n* (%)	*n* (%)	*p* Value	FIV vs. Control	FELV vs. Control	FIV vs. FELV
**Relapse**					0.285			
No	58 (52%)	27 (45%)	18 (62%)	13 (59%)		0.176	0.322	1.000
Yes	53 (48%)	33 (55%)	11 (38%)	9 (41%)				
**MTAS**					0.618			
No	33 (30%)	20 (33%)	8 (28%)	5 (23%)		0.634	0.426	0.756
Yes	78 (70%)	40 (67%)	21 (72%)	17 (77%)				
**Significant Improvement or Cure**					0.002 ^#^			
No	22 (20%)	6 (10%)	6 (51%)	10 (45%)		0.194	<0.001 *	0.074
Yes	89 (80%)	54 (90%)	23 (79%)	12 (55%)				

* p ≤ 0.017 was considered significant after Bonferroni correction for multiple comparisons. ^#^ *p* ≤ 0.05.

**Table 6:** Logistic regression models considering “No significant improvement” as the event of interest.

	Significant Improvement	No Significant Improvement	Model 1	Model 2	Model 3
	*n* (%)	*n* (%)	OR (95% CI)	OR (95% CI)	OR (95% CI)
**Group**					
Control	54 (90%)	6 (10%)	—	—	—
FIV	23 (79%)	6 (21%)	2.35 (0.67–8.27)	2.69 (0.73–10.1)	2.07 (0.57–7.47)
FELV	12 (55%)	10 (45%)	7.50 (2.35–26.1)	8.61 (2.28–37.3)	6.90 (2.07–25.0)
**Lingual Ulcer**					
No	68 (85%)	12 (15%)	—	—	
Yes	21 (68%)	10 (32%)	2.70 (1.01–7.17)	1.94 (0.64–5.65)	
**Proliferative Stomatitis**					
No	47 (80%)	12 (20%)	1.07 (0.42–2.79)	0.68 (0.21–2.06)	
Yes	42 (81%)	10 (19%)	—	—	
**Tooth Resorption**					
No	23 (77%)	7 (23%)	1.34 (0.46–3.61)	0.87 (0.25–2.75)	
Yes	66 (81%)	15 (19%)	—	—	
**Periodontal Disease**					
No	22 (79%)	6 (21%)	1.14 (0.37–3.17)	1.39 (0.40–4.59)	
Yes	67 (81%)	16 (19%)	—	—	
**Corticosteroids**					
No	49 (86%)	8 (14%)	—		—
Yes	40 (74%)	14 (26%)	2.14 (0.83–5.85)		2.17 (0.76–6.55)
**Surgery**					
Partial extraction	45 (85%)	8 (15%)	—		—
Full extraction	43 (75%)	14 (25%)	1.83 (0.71–5.00)		1.52 (0.52–4.56)
**No. of Phases**					
1	77 (79%)	20 (21%)	1.56 (0.38–10.5)		1.75 (0.37–12.7)
2	12 (86%)	2 (14%)	—		—
**Nagelkerke’s Pseudo R^2^**				0.20	0.20

Model 1: unadjusted OR; Model 2: adjusted for group and clinical variables; Model 3: adjusted for group and treatment variables; No. = number; “No significant improvement” = EDT 0 and 1; “Significant improvement” = EDT 2 and 3.

**Table 7:** Overall postsurgical survival time and mean age at time of death of the 111 cats with chronic gingivostomatitis, according with retroviral disease status.

	Control	FIV	FeLV
**Median Postsurgical Survival Time (Days)**	882	1425	346
**Mean Postsurgical Survival Time (Days)**	1291	1049	619
**Mean Age at Time of Death (Years)**	10.0 ± 4.2	9.6 ± 3.5	5.8 ± 3.6

## Data Availability

Data supporting the reported results can be sent to anyone interested by contacting the corresponding author.
